# A Novel Generalized Normal Distribution for Human Longevity and other Negatively Skewed Data

**DOI:** 10.1371/journal.pone.0037025

**Published:** 2012-05-18

**Authors:** Henry T. Robertson, David B. Allison

**Affiliations:** Department of Biostatistics, University of Alabama at Birmingham, Birmingham, Alabama, United States of America; National Taiwan University, Taiwan

## Abstract

Negatively skewed data arise occasionally in statistical practice; perhaps the most familiar example is the distribution of human longevity. Although other generalizations of the normal distribution exist, we demonstrate a new alternative that apparently fits human longevity data better. We propose an alternative approach of a normal distribution whose scale parameter is conditioned on attained age. This approach is consistent with previous findings that longevity conditioned on survival to the modal age behaves like a normal distribution. We derive such a distribution and demonstrate its accuracy in modeling human longevity data from life tables. The new distribution is characterized by 1. An intuitively straightforward genesis; 2. Closed forms for the pdf, cdf, mode, quantile, and hazard functions; and 3. Accessibility to non-statisticians, based on its close relationship to the normal distribution.

## Introduction

Variables with negatively skewed distributions can appear in situations where data cluster near an upper limit. Examples of such variables include human longevity [1], where most people in developed societies live to old age but few survive past age 100; the distribution of IQ scores [2]; in ectothermic animals, reproductive fitness as a function of body temperature [3]; and in medicine, the distribution of glomerular filtration rate in a population [4].

The methods for modeling such variables vary considerably. In the case of IQ, the skew is typically ignored and a normal distribution is imposed [5]–[6]. In other cases, reflections or power transformations are applied [7]–[8]. In still others, the variable is fitted to an extreme value distribution such as Weibull or Gompertz [9].

**Figure 1:**
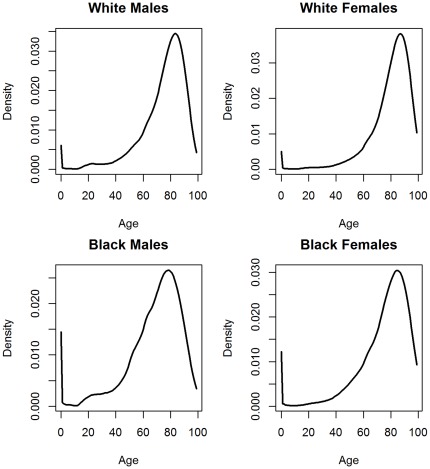
Density functions of life table data. The data for 2006 life tables were downloaded from the Centers for Disease Control (CDC).

We select human longevity as our motivating example, as it is a variable of great interest and importance with a long history of attempts to fit to a distribution. The distribution is characterized by 1. Strong negative skew; 2. Bimodality, with peaks at infancy and old age (Figure 1); and 3. Positive but finite values. Infant mortality is typically treated as a separate topic from adult mortality [10]. The former is driven by genetic errors, infectious diseases, or exposure, while the latter is driven by aging. Thus, parametric models for longevity generally exclude infant mortality; when necessary, mixture distributions are used to accommodate both.

Historically, adult longevity has been modeled with extreme value distributions. In 1825, the Gompertz distribution was proposed to model adult longevity [11]. In 1860, Makeham proposed a refinement, deriving the three-parameter Gompertz-Makeham distribution [12]. Since then, the Weibull distribution has sometimes been used for the same purpose, when the analysis is restricted to specific causes of death [13]. The Gompertz and Weibull distributions are specific cases of the generalized gamma or generalized extreme value distributions; the generalized distributions are occasionally used in survival analysis [14]. Extreme value distributions have an emphasis on rare events, such as the longest-lived individual, but longevity research is more often interested in group averages. Do other families of distributions offer alternatives?

In 2001, Kannisto [15] observed a relationship between longevity and the normal distribution. He described the importance of the distributional mode (M) as a consistent quantity for characterizing longevity: although life expectancy (as a mean) rose rapidly during the 20^th^ century due to decreases in infant mortality, the mode rose less. He also observed that longevity conditioned on survival past the mode was highly consistent with the behavior of a normal distribution: the ratio of the standard deviation above mode to life expectancy at mode was very close to 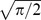 throughout different populations and time periods, with a correlation of +.995. Finally, he noted the effect of compression: over time, the right-hand slope has become increasingly vertical, corresponding to a decreasing SD(M+), as if meeting a resistance to further increases in the mode. These findings suggest that a good distribution for modeling human longevity could involve a Gaussian kernel that models compression past the mode. Are there other generalized normal distributions that already accomplish this?

Various generalizations of the normal distribution are in use. The most well-known among them appears to be that proposed by Nadarajah [16]; his version alters the kurtosis, adjusting the sharpness of the peak, but maintains a zero-skew symmetry. The inverse Gaussian distribution [17] is restricted to positive skew. The skew-normal distribution developed by Azzalini [18] does allow for negative skew, but has the constraint that skewness is limited to values between −1 and +1. Our survey of 74 life tables from around the world found that in 70 cases (95%), the sample skew of adult populations was less than −1. In our search, no generalized normal distributions explicitly addressed a compression of the scale parameter.

In the Methods section, we will derive a generalized normal distribution that builds upon Kannisto’s observations. In section 3, we will discuss its properties. In section 4, we will compare the fit of this distribution to three other distributions using life table data from around the world, and in section 5, we will offer a discussion.

## Methods

### 1. Genesis

**Figure 2:**
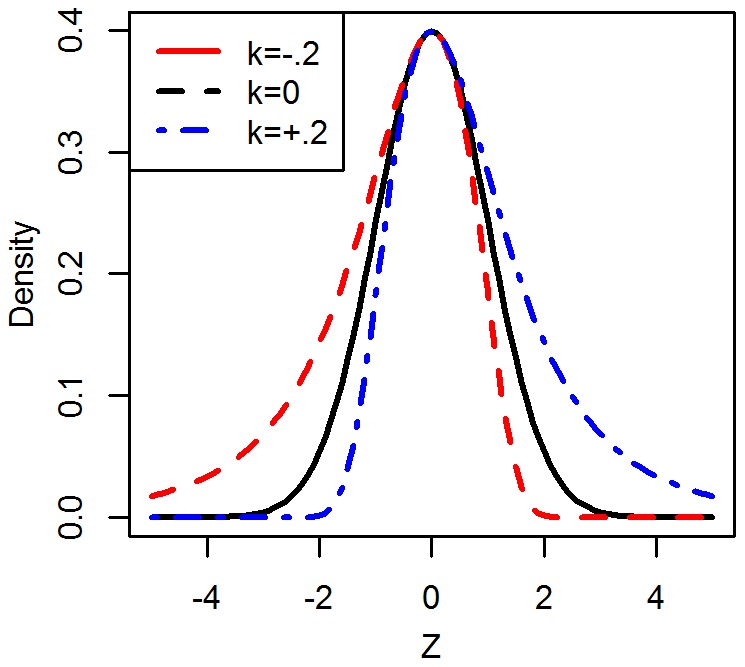
Plots of 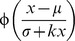, with μ  = 0 and σ  = 1.

Many distributions, including the normal, contain the location-scale transformation:
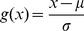
(1)The scale parameter in the denominator is a constant. One way to model compression (or expansion) is to condition the scale parameter on attained age. The function above can be altered as:
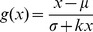
(2)When this function is applied to ϕ(•), the standard normal density, a skew is induced: when k is positive, a positive skew occurs; when k is negative, a negative skew occurs (Figure 2).

**Figure 3:**
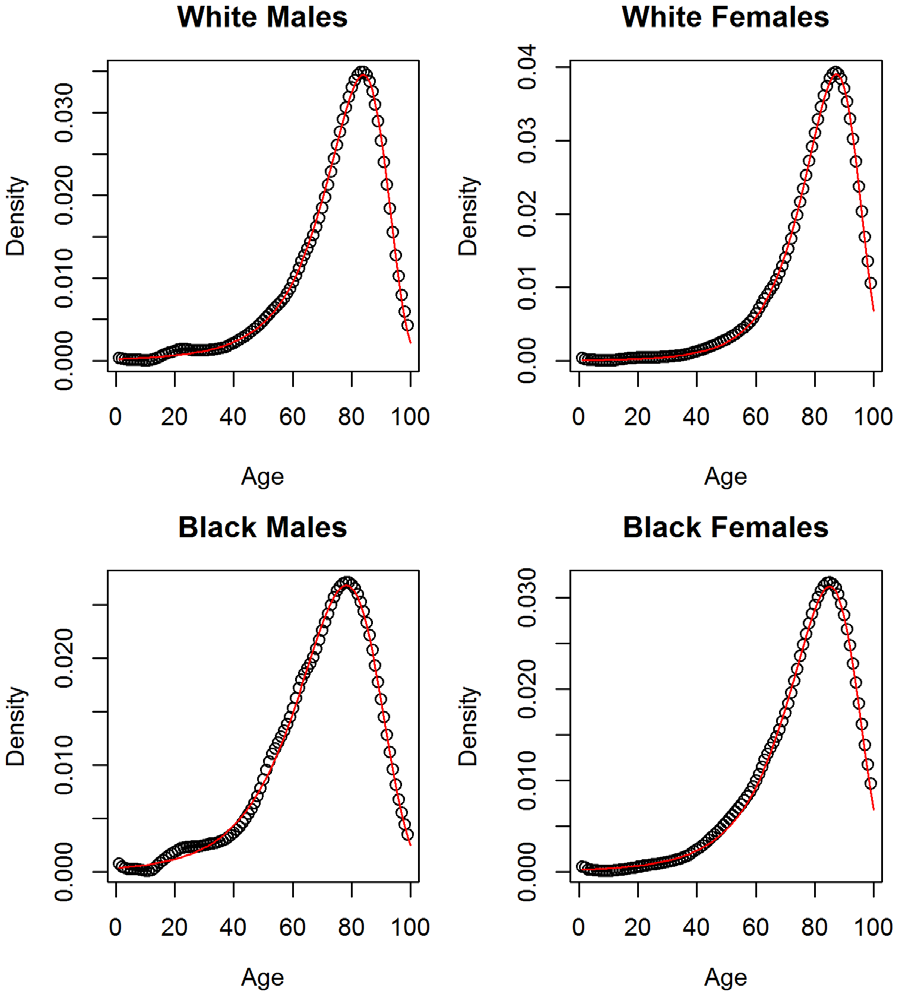
Fit of new distribution to life table data. Deaths at age 0 were excluded.

When restricted to negative skew, it is also possible to specify an equivalent parameterization of:
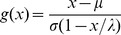
(3)Above, λ is an asymptotic upper bound of longevity and (1−x/λ) is the unspent portion of longevity at age x. This is equivalent to a normal distribution whose scale parameter decreases linearly with attained age. The normalized density is then derived as:
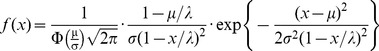
(4)This distribution was found to fit the observed density of US life table data well (Figure 3). We will denote this distribution as the compressed normal distribution to distinguish from other generalized normal distributions.

### 2. Properties of the Distribution

The distribution is supported on the domain (0, λ). All three parameters (μ, σ, λ) are restricted to positive values. We assumed 0< σ < μ < λ when deriving additional properties; such a constraint was found to hold for all life tables we examined.

Additional properties of the distribution are provided in Table 1. The detailed procedures for computing the mean and variance are provided in Appendices S1 and S2.

**Table 1:** Properties of the distribution.

Quantity	Formula
Parameters	0< σ < μ < λ
Domain	x 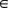 (0,λ)
PDF	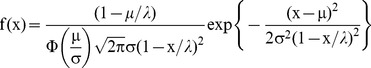
CDF	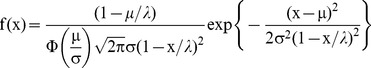
Hazard	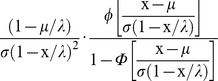
Quantile	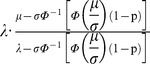
Median	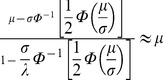
Mode	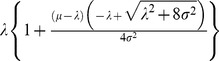
Mean	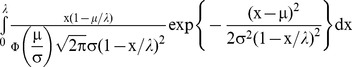
Variance	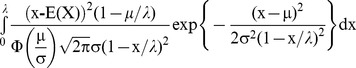

### 3. Transformation of a Normal Distribution

This distribution can also be viewed as a transformation of a truncated standard normal distribution. If Z is a standard normal distribution truncated on the left at –μ/σ, then the distribution is equivalent to:
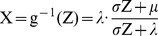
(5)


At age x, the distribution’s pdf, cdf, and hazard function is the same as Z at 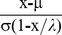.

### 4. Behavior of the Right Tail

**Figure 4:**
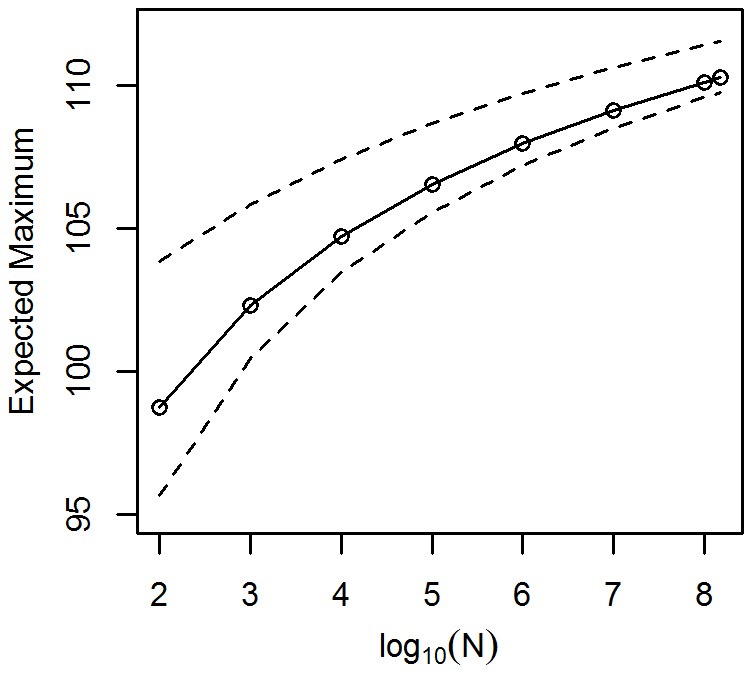
Estimated maximum longevity for a population N, based on parameter estimates for American white males. The dotted lines denote 95% confidence intervals. The parameter values were {μ, σ, λ}  =  {79.3, 32.8, 132.2}.

As the sample size approaches infinity, the maximum observed longevity will converge to λ. Since the normal distribution’s thin right tail is made even thinner by compression, the maximum will converge very slowly to λ. We illustrate this in Figure 4, using estimated parameter values for American white males. The means and confidence intervals were computed from the first-order statistic of a (0,1) uniform distribution, which were passed to the quantile function. Although the upper limit is λ  = 135, the expected first-order value for a population of 150 million is 110.3. This corresponds closely to real-world data, where the oldest living American male as of this writing is Shelby Harris, at 110 years of age [19].

### 5. The Hazard Function

**Figure 5:**
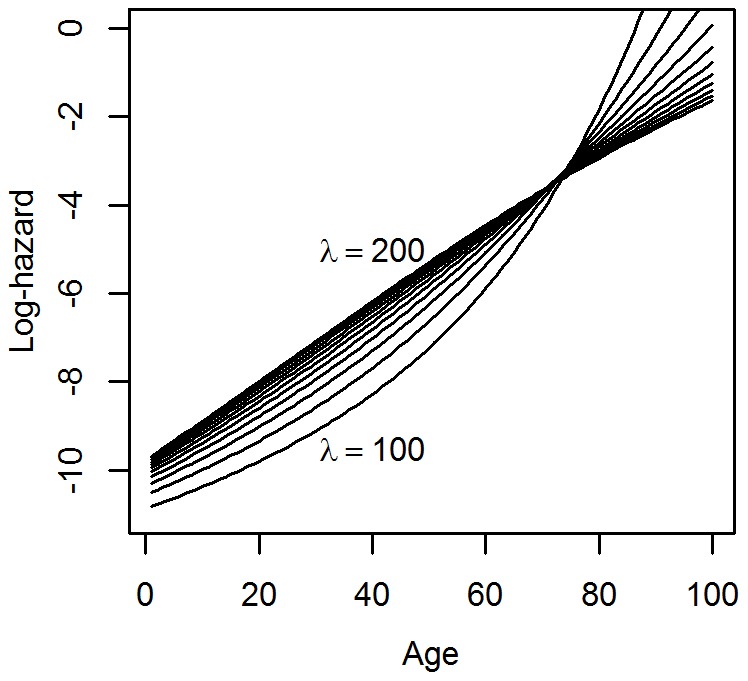
Sample graphs of log-hazard functions. Here, μ  = 80, σ  = 25, and λ varied from 100 to 200.

Some sample graphs of log-hazard functions are shown in Figure 5. As apparent from the graph, this distribution can model log-hazard rates that accelerate, increase linearly, or decelerate. There are arguments that hazard rates decelerate in very old age [20], although the author of the CDC’s 2006 life tables found no evidence to support this notion [21]. The CDC author states that the purported leveling of the hazard rate was likely an artifact of age misreporting among the very old; this debate remains an open topic.

Survival analysis makes extensive use of hazard rates. One known drawback of modeling survival based on hazard rates is frailty: those who survive to old age come from an increasingly homogeneous pool of survivors, thus estimates of the hazard function become biased [22]. The compressed normal distribution offers one way to model the increasing homogeneity of the population as it ages, and may improve the accuracy of estimated hazard rates in future survival analyses; this topic will be explored in further papers.

### 6. Application to Life Tables

We compared the fit of the compressed normal distribution against other distributions using life table data from multiple countries. We fitted the two-parameter Gompertz distribution as a reference, and then compared the AIC (Akaike Information Criterion) scores.

### 7. Selection of Life Tables

An excerpt from United States life table data is shown in Table 2. Life tables for countries other than the United States were downloaded from the Human Life-Table Database [23]. In order for the life tables to meet sufficient standards of quality for this analysis, the following restrictions were applied:

1. Availability of complete data in one-year increments.
2. Availability of data to at least age 90, well past the mode.

**Table 2:** Excerpt from the 2006 CDC life tables.

	Probablity of dying between ages x to x +1	Number surviving to age x	Number dying between ages x to x +1	Person-years lived between ages x to x +1	Total number of person-years lived above age x	Expectation of life at age x
Age	q_x_	l_x_	d_x_	L_x_	T_x_	e_x_
0–1	0.006119	100,000	612	99,462	7,566,361	75.7
1–2	0.000398	99,388	40	99,368	7,466,899	75.1
2–3	0.000296	99,349	29	99,334	7,367,531	74.2
3–4	0.000227	99,319	22	99,308	7,268,197	73.2
4–5	0.000182	99,297	18	99,288	7,168,889	72.2
5–6	0.000171	99,279	17	99,270	7,069,601	71.2
6–7	0.000161	99,262	16	99,254	6,970,331	70.2
7–8	0.000148	99,246	15	99,238	6,871,078	69.2
8–9	0.000127	99,231	13	99,225	6,771,839	68.2
9–10	0.000100	99,218	10	99,213	6,672,615	67.3

We found 74 life tables from 35 countries that met the above criteria. From these countries, the most recent life tables were selected. We excluded deaths before age 3, as the outcome of interest was adult longevity.

**Table 3:** AIC Regression Results.

Variable	Coefficient	SE	p-value
Intercept (Gompertz)	750,480.0	2,287.6	<.0001
Compressed Normal	−1,936.5	891.6	0.0305
Gompertz-Makeham	−1,186.3	891.6	0.1842
Generalized Gamma	2,580.3	891.6	0.0040
Skew-Normal	3,056.3	891.6	0.0007
Gen. Extreme Value	16,793.8	891.6	<.0001
factor(country/sex)Australia M	25,761.5	3,131.1	<.0001
factor(country/sex)Austria F	−23,508.1	3,131.1	<.0001
…			

**Table 4:** Results of varying initial parameter values.

Initial Values			Distance	Final Values		
μ_0_	σ_0_	λ_0_		μ	σ	λ
79.3	32.8	132.2	0.0	79.33128	32.83232	132.1717
69.3	32.8	132.2	10.0	79.33128	32.83232	132.1717
89.3	32.8	132.2	10.0	79.33128	32.83232	132.1717
64.3	32.8	132.2	15.0	79.33128	32.83232	132.1717
94.3	32.8	132.2	15.0	79.33128	32.83232	132.1717
79.3	12.8	132.2	20.0	79.33128	32.83232	132.1717
79.3	52.8	132.2	20.0	79.33128	32.83232	132.1717
79.3	32.8	102.2	30.0	79.33128	32.83232	132.1717
79.3	32.8	162.2	30.0	79.33128	32.83232	132.1717
75.0	25.0	100.0	33.4	79.33128	32.83232	132.1717
75.0	45.0	100.0	34.7	79.33128	32.83232	132.1717
65.0	45.0	100.0	37.3	DNC		
70.0	45.0	100.0	35.6	DNC		
75.0	45.0	105.0	30.1	DNC		
75.0	35.0	105.0	27.6	79.33128	32.83232	132.1717
95.0	35.0	105.0	31.4	DNC		
95.0	35.0	110.0	27.2	79.33128	32.83232	132.1717

DNC  =  Did not converge. “Distance” was defined as the Euclidean distance between the initial values and final estimates.

### 8. Model

The life table data were fitted against five distributions: Gompertz, Gompertz-Makeham, compressed normal, Azzalini skew-normal, generalized gamma, and generalized extreme value.

The distributions’ parameters were estimated according to the least squares method [24] of minimizing the SSE, i.e.:
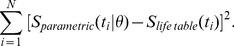
(6)We fitted the conditional probability of surviving to age x given that they survived to age 3. Using nonlinear least squares, we fitted the regression equation:
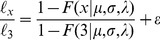
(7)The above was implemented through procedure nls in R 2.14.0. The parameter estimates were then applied to likelihood equations for the life table cohorts of 10,000 hypothetical subjects in order to derive the AIC. Deaths before age 3 were excluded. Finally, we compared the AIC scores using ordinary least squares. The outcome variable was AIC, and the predictor variables were distribution type and life table type. Technical details are provided in Appendix S3.

**Figure 6:**
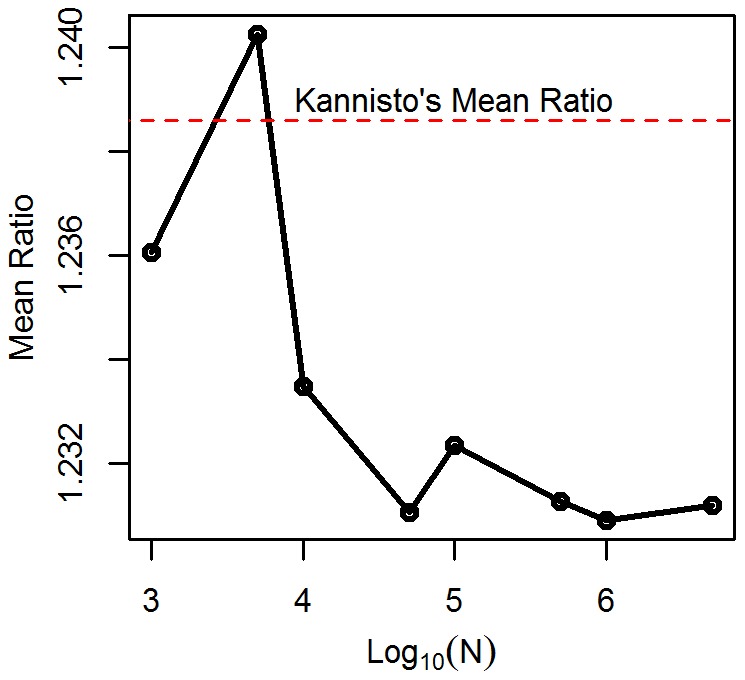
Simulation results based on 480 hypothetical populations with 500,000 members each. The slope of 1.2295 was closer to Kannisto’s estimates than 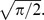

Nonlinear least squares require the specification of initial values for parameter estimates. As a secondary analysis, we varied the initial values to determine its effect on final estimates. This was done using data from the life table for white females in the United States.

## Results

In 46 out of 74 life tables (62%), the compressed normal distribution provided the best fit as judged by AIC (Table 3). The average AIC for a life table fit with the two-parameter Gompertz distribution was 750,480. The generalized gamma, skew-normal, and generalized extreme value distributions had significantly higher AIC scores. The Gompertz-Makeham distribution had a lower average score than Gompertz, but was not statistically significant. The compressed normal distribution was significantly lower by an average of 1,937 points. Whether we adjusted for individual life tables as fixed effects or random effects, the results were identical within 4 significant figures. Detailed results including the parameter estimates are provided in Appendix S4.

When we varied the initial values supplied to the model, the final estimates were identical for all cases when the model converged (Table 4). This suggests that the identifiability of parameter estimates is not a major problem with this distribution. The μ, σ, and λ parameters respectively tolerated misspecifications of up to 15, 20, and 30 from their “true” values.

## Discussion

Our preliminary demonstration made use of default settings in R’s nls procedure, which invoked the Gauss-Newton algorithm. Potentially, all of the distributions could have achieved better fits with more sophisticated algorithms, though we did not wish to make it the focus of this paper. Nevertheless, we did demonstrate the accessibility of good estimates for the compressed normal distribution without resorting to advanced programming. In future papers, we will explore more details of finding estimates and their variances.

The good fit of the distribution came at the expense of two problems: 1. the mean is an infinite sequence; and 2. the normal equations lack closed-form solutions, as the three parameters depend on each other’s values. We have addressed limitation #1 by providing software that automatically computes the mean. When working with longevity, medians are generally preferred over means. The median is closely approximated by the value of μ (within 0.1); exact values can also be computed using the formula provided. Changes in μ can be understood as changes in the median.

For limitation #2, we have found that the parameters exhibited unimodal likelihood properties, making estimation straightforward. The use of gradient functions led to rapid convergence when using nonlinear optimization software; we will elaborate on this in a future paper. For the purposes of this demonstration, we supplied initial parameter estimates of {μ, σ, λ}  =  {80,24,140}. The support of the distribution depends on the parameter λ, but we did not encounter difficulties in estimability; λ lies well outside the range of observed values. The secondary analysis found that the final estimates were robust to misspecification, yielding identical estimates whenever the model converged.

As a final check, we compared the characteristics of this distribution to Kannisto’s observation that the ratio of the standard deviation above mode to life expectancy at mode was very close to 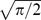 ≈ 1.2533 across life tables. Kannisto’s ratios were slightly below this value, with a mean value of 1.2386 and an SD of 0.0112 (Table 1 of [15]). We ran simulations based on the estimated parameter values for life tables from Table 4. We varied the sample size from 1,000 to 5 million. At the smaller sample sizes, the mean ratio straddled Kannisto's values (Figure 6); however, at larger sample sizes, it appeared to converge toward a lower value of 1.231. We will investigate the nature of this possibly novel constant in future papers.

The compressed normal distribution shows promise as a model for human longevity, particularly survival analysis. Even today, the semi-parametric Cox model is still preferred over parametric models when conducting survival analyses, due to small but consistent discrepancies between estimated and empirical values [25]. Parametric models, when accurate, offer the advantage of directly estimating changes in average life expectancies. Additionally, parametric models can estimate median longevity even when the censoring rate is above 50%.

In future work, we will develop methods for parametric survival analysis using this distribution to determine the association of BMI and other chronic disease risk factors with longevity at the population level. Additionally, we believe this distribution is not only useful for modeling human longevity, but also other variables with skewed distributions.

## Supporting Information

Appendix S1 **Deriving the mean.**
(DOCX)

Appendix S2 **Deriving the Variance.**
(DOCX)

Appendix S3 **Fitting life table data to nonlinear least squares in R.**
(DOCX)

Appendix S4 **Detailed results of distribution fits.**
(DOCX)
