# Correction: A Novel Generalized Normal Distribution for Human Longevity and other Negatively Skewed Data

**DOI:** 10.1371/journal.pone.0151123

**Published:** 2016-03-03

**Authors:** Henry T. Robertson, David B. Allison

In Table 1, the “Formula” column for “CDF” is incorrect. Please see the corrected Table 1 here.

**Table 1:** Properties of the distribution.

Quantity	Formula
Parameters	0 < σ < μ < λ
Domain	x є (0,λ)
PDF	$\text{f}(\text{x})=\frac{\left(1-\mu /\lambda \right)}{\Phi \left(\frac{\mu }{\sigma }\right)\sqrt{2\pi }\sigma {\left(1-\text{x}/\lambda \right)}^{2}}\text{exp}\left\{-\frac{{\left(\text{x}-\mu \right)}^{2}}{{2\sigma }^{2}{\left(1-\text{x}/\lambda \right)}^{2}}\right\}$
CDF	$\text{F}(\text{x})=1-\frac{1-\Phi \left(\frac{\text{x}-\mu }{\sigma (1-\text{x}/\lambda )}\right)}{\Phi \left(\frac{\mu }{\sigma }\right)}$
Hazard	$\frac{\left(1\;-\;\mu /\lambda \right)}{\sigma {\left(1-\text{x}/\lambda \right)}^{2}}\cdot \frac{\phi \left[\frac{\text{x}-\mu }{\sigma (1-\text{x}/\lambda )}\right]}{1-\Phi \left[\frac{\text{x}-\mu }{\sigma (1-\text{x}/\lambda )}\right]}$
Quantile	$\lambda \cdot \frac{\mu -{\sigma \Phi }^{-1}\left[\Phi \left(\frac{\mu }{\sigma }\right)(1-\text{p})\right]}{\lambda -{\sigma \Phi }^{-1}\left[\Phi \left(\frac{\mu }{\sigma }\right)(1-\text{p})\right]}$
Median	$\frac{\mu -\sigma \Phi^{-1}\left[\frac{1}{2}\Phi \left(\frac{\mu }{\sigma }\right)\right]}{1-\frac{\sigma }{\lambda }\Phi^{-1}\left[\frac{1}{2}\Phi \left(\frac{\mu }{\sigma }\right)\right]}\approx \mu$
Mode	$\lambda \left\{1+\frac{\left(\mu -\lambda )(-\lambda +\sqrt{\lambda^{2}+8\sigma^{2}}\right)}{4\sigma^{2}}\right\}$
Mean	$\int_{0}^{\lambda }\frac{\text{x}(1-\mu /\lambda )}{\Phi \left(\frac{\mu }{\sigma }\right)\sqrt{2\pi }\sigma (1-\text{x}/\lambda {)}^{2}}\text{exp}\left\{-\frac{{\left(\text{x}-\mu \right)}^{2}}{{2\sigma }^{2}{\left(1-\text{x}/\lambda \right)}^{2}}\right\}\text{dx}$
Variance	$\int_{0}^{\lambda }\frac{{\left(\text{x}-\text{E}(\text{X})\right)}^{2}(1-\mu /\lambda )}{\Phi \left(\frac{\mu }{\sigma }\right)\sqrt{2\pi }\sigma (1-\text{x}/\lambda {)}^{2}}\text{exp}\left\{-\frac{{\left(\text{x}-\mu \right)}^{2}}{{2\sigma }^{2}{\left(1-\text{x}/\lambda \right)}^{2}}\right\}\text{dx}$

## References

[1] Robertson HT, Allison DB (2012) A Novel Generalized Normal Distribution for Human Longevity and other Negatively Skewed Data. PLoS ONE 7(5): e37025 doi: 10.1371/journal.pone.0037025 22623974 10.1371/journal.pone.0037025 PMC3356396

