# Supplementary material for: A Novel Generalized Normal Distribution for Human Longevity and other Negatively Skewed Data
Source: PLoS One. 2012 May 18;7(5):e37025. doi: 10.1371/journal.pone.0037025 (PMC3356396; doi:10.1371/journal.pone.0037025)
Supplement: Appendix S1 — Deriving the mean. (DOCX) [file pone.0037025.s001.docx]

**Appendix S1: Deriving the mean.**

To compute the expectation E(X), a Taylor series expansion was used.

$$f(x)=\frac{1}{\sqrt{2\pi}\Phi\left(\frac{\mu}{\sigma}\right)}\cdot\frac{(\text{1-μ/λ)}}{\sigma(1-x/\lambda)^{2}}\cdot\exp\left\{-\frac{(x-\mu)^{2}}{2\sigma^{2}(1-x/\lambda)^{2}}\right\}$$

$$w=\frac{x-\mu}{\sigma(1-x/\lambda)}\Leftrightarrow x=\frac{\lambda(\sigma w+\mu)}{\sigma w+\lambda}$$

Taylor series expansion:

$$g'(w)=\frac{(\sigma w+\lambda)\lambda\sigma-\lambda(\sigma w+\mu)\sigma}{(\sigma w+\lambda)^{2}}=\frac{\lambda\sigma(\lambda+\sigma w-\sigma w-\mu)}{(\sigma w+\lambda)^{2}}=\frac{\lambda\sigma(\lambda-\mu)}{(\sigma w+\lambda)^{2}}$$

$$g''(w)=\frac{-2\lambda\sigma^{2}(\lambda-\mu)}{(\sigma w+\lambda)^{3}}$$

$$g'''(w)=\frac{3!\lambda\sigma^{3}(\lambda-\mu)}{(\sigma w+\lambda)^{4}}$$

$$g^{(n)}(w)=\frac{\lambda(-1)^{n+1}\sigma^{n-1}n!(\lambda-\mu)}{(\sigma w+\lambda)^{n+1}}$$

$$g(0)=\frac{\lambda(0\sigma+\mu)}{\lambda+0\sigma}=\mu$$

$$g'(0)=\frac{\lambda\sigma(\lambda-\mu)}{(\lambda+0\sigma)^{2}}=\frac{\lambda\sigma(\lambda-\mu)}{\lambda^{2}}=\frac{\sigma(\lambda-\mu)}{\lambda}=\frac{\sigma}{\lambda}(\lambda-\mu)$$

$$g''(0)=\frac{-2\lambda\sigma^{2}(\lambda-\mu)}{(\lambda+0\sigma)^{3}}=\frac{-2\lambda\sigma^{2}(\lambda-\mu)}{\lambda^{3}}=\frac{-2\sigma^{2}(\lambda-\mu)}{\lambda^{2}}=-2\frac{\sigma^{2}}{\lambda^{2}}(\lambda-\mu)$$

$$g'''(0)=\frac{3!\lambda\sigma^{3}(\lambda-\mu)}{(\lambda+0\sigma)^{4}}=\frac{3!\lambda\sigma^{3}(\lambda-\mu)}{\lambda^{4}}=\frac{3!\sigma^{3}(\lambda-\mu)}{\lambda^{3}}=3!\frac{\sigma^{3}}{\lambda^{3}}(\lambda-\mu)$$

$$g^{(n)}(0)=n!(-1)^{n+1}\frac{\sigma^{n}}{\lambda^{n}}(\lambda-\mu)$$

The Maclaurin series is thus

$$\begin{matrix} =\mu+\frac{\sigma}{\lambda}(\lambda-\mu)w-\frac{\sigma^{2}}{\lambda^{2}}(\lambda-\mu)w^{2}+\frac{\sigma^{3}}{\lambda^{3}}(\lambda-\mu)w^{3}-... \\ =\mu-(\lambda-\mu)\sum_{i=1}^{\infty} \left(-\frac{\sigma}{\lambda}w\right)^{i} \end{matrix}$$

Then

$E(X)=\frac{1}{\Phi\left(\frac{\mu}{\sigma}\right)}\int_{\frac{-\mu}{\sigma}}^{\infty} g(w)\varphi(w)dw$ $=\frac{1}{\Phi\left(\frac{\mu}{\sigma}\right)}\int_{-\frac{\mu}{\sigma}}^{\infty} \left[\mu+\frac{\sigma}{\lambda}(\lambda-\mu)w-\frac{\sigma^{2}}{\lambda^{2}}(\lambda-\mu)w^{2}+\frac{\sigma^{3}}{\lambda^{3}}(\lambda-\mu)w^{3}-...\right]\varphi(w)dw$

$$=\frac{\lambda-\mu}{\Phi\left(\frac{\mu}{\sigma}\right)}\int_{-\frac{\mu}{\sigma}}^{\infty} \left[\frac{\mu}{\lambda-\mu}+\frac{\sigma}{\lambda}w-\frac{\sigma^{2}}{\lambda^{2}}w^{2}+\frac{\sigma^{3}}{\lambda^{3}}w^{3}-...\right]\varphi(w)dw$$

Lemma 1:

$$\int_{a}^{\infty} \varphi(w)dw=1-\Phi(a)$$

Lemma 2:

$$\int_{a}^{\infty} w\varphi(w)dw=\left.-\frac{1}{\sqrt{2\pi}}\exp\left\{-\frac{w^{2}}{2}\right\}\right|_{a}^{\infty}=-\frac{1}{\sqrt{2\pi}}\left[e^{-\frac{\infty^{2}}{2}}-e^{-\frac{a^{2}}{2}}\right]=\varphi(a)$$

Lemma 3:

$$\int_{a}^{\infty} w^{2}\varphi(w)dw=\int_{a}^{\infty} w\left[w\cdot\frac{1}{\sqrt{2\pi}}\exp\left\{-\frac{w^{2}}{2}\right\}\right]dw=\left.-w\varphi(w)\right|_{a}^{\infty}+\int_{a}^{\infty} \varphi(w)dw$$

$$=a\varphi(a)+1-\Phi(a)$$

Lemma 4:

$$\begin{matrix} \int_{a}^{\infty} w^{n}\varphi(w)dw=\int_{a}^{\infty} w^{n-1}[w\varphi(w)]dw=-[w^{n-1}\varphi(w)]_{a}^{\infty}+(n-1)\int_{a}^{\infty} w^{n-2}\varphi(w)dw \\ =a^{n-1}\varphi(a)+(n-1)E(w^{n-2}) \end{matrix}$$

Define

$$\begin{matrix} a=-\frac{\mu}{\sigma},b=\frac{\sigma}{\lambda},k=\frac{\lambda-\mu}{\Phi(a)} \\ e_{0}=1-\Phi(a) \\ e_{1}=\varphi(a) \\ e_{2}=a\varphi(a)+e_{0} \\ e_{3}=a^{2}\varphi(a)+2e_{1} \\ e_{4}=a^{3}\varphi(a)+3e_{2} \\ e_{n}=a^{n-1}\varphi(a)+(n-1)e_{n-2} \end{matrix}$$

Then

$$E(X)=\mu+k(be_{1}-b^{2}e_{2}+b^{3}e_{3}-b^{4}e_{4}+b^{5}e_{5}-b^{6}e_{6}+...)$$

This sequence will converge if b < 1, i.e. σ < λ. If this constraint is not met, then the mean can be estimated by numerical simulation.

Simulations revealed that expansion to e_6_ was found to yield estimates within 0.1 of the sample means.
