# Supplementary material for: A Novel Generalized Normal Distribution for Human Longevity and other Negatively Skewed Data
Source: PLoS One. 2012 May 18;7(5):e37025. doi: 10.1371/journal.pone.0037025 (PMC3356396; doi:10.1371/journal.pone.0037025)
Supplement: Appendix S2 — Deriving the Variance. (DOCX) [file pone.0037025.s002.docx]

**Appendix S2: Deriving the Variance.**

Similarly, E(X^2^) was found by a Taylor series expansion.

$$x^{2}=\frac{\lambda^{2}(\sigma w+\mu)^{2}}{(\sigma w+\lambda)^{2}}=h(w)$$

Taylor series expansion of h(w):

$$h'''(w)=(\lambda-\mu)\sigma^{3}\lambda^{2}[-4!(\lambda-\mu)(\sigma w+\lambda)^{-5}+2\cdot3!(\sigma w+\lambda)^{-4}]$$

$$h^{(n)}(w)=(\lambda-\mu)\lambda^{2}\sigma^{n}[(\lambda-\mu)(n+1)!(-1)^{n}(\sigma w+\lambda)^{-n-2}+2\cdot n!(-1)^{n+1}(\sigma w+\lambda)^{-n-1}]$$

$$h(0)=\frac{\lambda^{2}\mu^{2}}{\lambda^{2}}=\mu^{2}$$

$$h'(0)=2\sigma\lambda^{2}(\lambda-\mu)\frac{\mu}{\lambda^{3}}=2\frac{\sigma}{\lambda}(\lambda-\mu)\mu=2(\lambda-\mu)b\mu$$

$$\begin{matrix} h^{(n)}(0)=(\lambda-\mu)^{2}\lambda^{-n}\sigma^{n}(n+1)!(-1)^{n}+2\cdot n!\cdot(\lambda-\mu)\lambda^{-n+1}\sigma^{n}(-1)^{n+1} \\ =(\lambda-\mu)^{2}b^{n}(n+1)!(-1)^{n}+2(\lambda-\mu)\lambda b^{n}n!(-1)^{n+1} \\ =(\lambda-\mu)b^{n}[(\lambda-\mu)(n+1)!(-1)^{n}+2\lambda n!(-1)^{n+1}] \end{matrix}$$

The Maclaurin series is thus

$$=\mu^{2}+2(\lambda-\mu)b\mu w+...+(\lambda-\mu)[(\lambda-\mu)(n+1)(-1)^{n}+2\lambda(-1)^{n+1}]b^{n}w^{n}+...$$

$$=\mu^{2}+(\lambda-\mu)\{2b\mu w+...+[(\lambda-\mu)(n+1)(-1)^{n}+2\lambda(-1)^{n+1}]b^{n}w^{n}+...\}$$

$$E(X^{2})=\frac{1}{\Phi\left(\frac{\mu}{\sigma}\right)}\int_{\frac{-\mu}{\sigma}}^{\infty} h(w)\varphi(w)dw$$

$$=\frac{1}{\Phi\left(\frac{\mu}{\sigma}\right)}\int_{-\frac{\mu}{\sigma}}^{\infty} \{\mu^{2}+(\lambda-\mu)\{2b\mu w+...+[(\lambda-\mu)(n+1)(-1)^{n}+2\lambda(-1)^{n+1}]b^{n}w^{n}+...\}\}\varphi(w)dw$$

$$=\mu^{2}+\frac{(\lambda-\mu)}{\Phi\left(\frac{\mu}{\sigma}\right)}\int_{-\frac{\mu}{\sigma}}^{\infty} \{2b\mu w+...+[(\lambda-\mu)(n+1)(-1)^{n}+2\lambda(-1)^{n+1}]b^{n}w^{n}+...\}\varphi(w)dw$$

$$E(X^{2})=\mu^{2}+k\{2b\mu e_{1}+...+[(\lambda-\mu)(n+1)(-1)^{n}+2\lambda(-1)^{n+1}]b^{n}e_{n}+...\}$$

$$Var(X)=E(X^{2})-E(X)^{2}$$

Simulations revealed that expanding E(X^2^) and E(X)^2^ to 21 terms found a variance estimate within approximately 0.1 of the median of sample variances.
