# Supplementary material for: A Novel Generalized Normal Distribution for Human Longevity and other Negatively Skewed Data
Source: PLoS One. 2012 May 18;7(5):e37025. doi: 10.1371/journal.pone.0037025 (PMC3356396; doi:10.1371/journal.pone.0037025)
Supplement: Appendix S3 — Fitting life table data to nonlinear least squares in R. (DOCX) [file pone.0037025.s003.docx]

**Appendix S3: Fitting life table data to nonlinear least squares in R.**

# Read data for Australian males

library(sn); library(VGAM); library(gamlss); library(eha)

source(“robertson_surv.R”)

w = read.table(“australia.txt”, sep = "\t", header=T, na.strings = c("."))

# Define minimum age

minimum = 3

# Select males only, 3 or older.

w = w[w$Sex==1 & w$Age>=minimum,]

# Define survival probability as (# alive at age x) / (# alive at age 18)

w$s = w$l/w[w$Age==18,]$l

# Create cohort data vector, for later computation of likelihood score

tempdata = rep(ww$Age, ww$d)

# Fit Compressed Normal distribution

r = nls(s ~ (1 - probertson(Age, mu, sigma, lambda))/(1 - probertson(18, mu, sigma, lambda)), data=w, start = list(mu = 85, sigma = 25, lambda = 135))

summary(r)

# Find LL

sum(drobertson(tempdata, coef(r)[1], coef(r)[2], coef(r)[3], log=TRUE))

# Fit Gompertz-Makeham distribution

r = nls(s ~ (1 - pmakeham(Age, shape=c(exp(-shape1), exp(-shape2)), scale=scale))/(1 - pmakeham(18, shape=c(exp(-shape1), exp(-shape2)), scale=scale)),

data=w, start = list(shape1 = 12, shape2 = 7, scale=9))

summary(r)

sum(dmakeham(tempdata, coef(r)[1], coef(r)[2], coef(r)[3], log=TRUE))

# Fit Azzalini distribution

r = nls(s ~ (1 - psn(Age, location, scale, shape))/(1 - psn(18, location, scale, shape)), data=w, start = list(location = 96, scale = 20, shape = -6))

summary(r)

sum(dsn(tempdata, coef(r)[1], coef(r)[2], coef(r)[3], log=TRUE))

# Fit Generalized Gamma distribution

r = nls(s ~ (1 - pGG(Age, mu, sigma, lambda))/(1 - pGG(18, mu, sigma, lambda)), data=w, start = list(mu = 87, sigma = .11, lambda = 15))

summary(r)

sum(dGG(tempdata, coef(r)[1], coef(r)[2], coef(r)[3], log=TRUE))

# Fit Generalized Extreme Value distribution

r = nls(s ~ (1 - pgev(Age, mu, sigma, lambda))/(1 - pgev(18, mu, sigma, lambda)), data=w, start = list(mu = 75, sigma = 16, lambda = -.5))

summary(r)

sum(dgev(tempdata, coef(r)[1], coef(r)[2], coef(r)[3], log=TRUE))

# Repeat above for other nations/sexes.
