# Supplementary material for: A Novel Generalized Normal Distribution for Human Longevity and other Negatively Skewed Data
Source: PLoS One. 2012 May 18;7(5):e37025. doi: 10.1371/journal.pone.0037025 (PMC3356396; doi:10.1371/journal.pone.0037025)
Supplement: Appendix S4 — Detailed results of distribution fits. (DOCX) [file pone.0037025.s004.docx]

**Appendix S4: Detailed results of distribution fits.**

| **Country** | **Sex** | **μ** | **σ** | **λ** | **SSE** | **Mode** | **LL** | **AIC** |
| --- | --- | --- | --- | --- | --- | --- | --- | --- |
| Australia | M | 82.49 | 31.02 | 129.16 | 0.30 | 86.90 | -386,200 | 772,407 |
| Australia | F | 86.71 | 30.40 | 127.42 | 0.29 | 90.52 | -372,151 | 744,307 |
| Austria | M | 80.56 | 30.35 | 132.14 | 0.31 | 85.09 | -387,115 | 774,237 |
| Austria | F | 85.82 | 31.40 | 122.13 | 0.26 | 89.66 | -364,192 | 728,390 |
| Belgium | M | 80.66 | 30.66 | 131.83 | 0.20 | 85.24 | -390,969 | 781,943 |
| Belgium | F | 85.95 | 32.05 | 125.27 | 0.43 | 90.08 | -378,827 | 757,661 |
| Canada | M | 80.05 | 28.58 | 136.83 | 0.21 | 84.29 | -390,978 | 781,962 |
| Canada | F | 84.98 | 29.03 | 136.31 | 0.22 | 88.95 | -384,178 | 768,363 |
| Chile | M | 75.44 | 27.43 | 154.29 | 0.05 | 79.88 | -399,662 | 799,331 |
| Chile | F | 81.81 | 28.44 | 143.63 | 0.06 | 86.02 | -389,906 | 779,818 |
| China | M | 72.77 | 30.48 | 126.39 | 0.70 | 77.87 | -378,944 | 757,894 |
| China | F | 76.09 | 33.64 | 124.24 | 0.63 | 81.62 | -373,877 | 747,760 |
| Czech | M | 76.64 | 26.95 | 145.20 | 0.43 | 80.81 | -395,175 | 790,355 |
| Czech | F | 82.95 | 30.10 | 122.37 | 0.24 | 86.83 | -372,174 | 744,354 |
| Denmark | M | 79.49 | 28.90 | 133.94 | 0.17 | 83.79 | -388,825 | 777,655 |
| Denmark | F | 83.49 | 28.06 | 136.44 | 0.16 | 87.34 | -380,878 | 761,762 |
| Finland | M | 79.67 | 32.71 | 131.21 | 0.33 | 84.85 | -397,094 | 794,193 |
| Finland | F | 85.98 | 32.20 | 122.52 | 0.40 | 89.99 | -373,306 | 746,617 |
| France | M | 80.30 | 32.49 | 133.10 | 0.37 | 85.43 | -397,777 | 795,560 |
| France | F | 87.15 | 33.29 | 124.24 | 0.45 | 91.34 | -378,267 | 756,540 |
| Germany | M | 80.06 | 28.32 | 138.11 | 0.20 | 84.26 | -390,127 | 780,259 |
| Germany | F | 85.26 | 30.20 | 125.35 | 0.32 | 89.07 | -372,674 | 745,354 |
| Greece | M | 79.49 | 29.28 | 134.09 | 0.38 | 83.90 | -393,009 | 786,024 |
| Greece | F | 83.55 | 25.53 | 127.03 | 0.89 | 86.59 | -370,257 | 740,519 |
| Hong Kong | M | 81.86 | 26.29 | 145.90 | 0.23 | 85.55 | -388,495 | 776,996 |
| Hong Kong | F | 87.82 | 26.62 | 141.31 | 0.34 | 91.16 | -373,356 | 746,718 |
| Iceland | M | 82.44 | 29.02 | 128.27 | 0.34 | 86.36 | -376,954 | 753,913 |
| Iceland | F | 85.43 | 27.34 | 133.32 | 0.20 | 88.89 | -366,888 | 733,783 |
| Ireland | M | 79.93 | 29.52 | 128.76 | 0.40 | 84.20 | -388,144 | 776,295 |
| Ireland | F | 84.38 | 28.83 | 130.06 | 0.37 | 88.16 | -378,810 | 757,627 |
| Israel | M | 81.90 | 28.53 | 135.53 | 0.07 | 85.96 | -385,594 | 771,194 |
| Israel | F | 85.27 | 28.79 | 127.14 | 0.05 | 88.86 | -369,688 | 739,381 |
| Italy | M | 81.64 | 29.05 | 131.78 | 0.23 | 85.74 | -386,194 | 772,394 |
| Italy | F | 86.73 | 29.13 | 127.80 | 0.38 | 90.29 | -372,347 | 744,699 |
| Japan | M | 82.10 | 29.34 | 136.37 | 0.21 | 86.36 | -390,819 | 781,643 |
| Japan | F | 88.86 | 30.76 | 130.32 | 0.47 | 92.67 | -376,241 | 752,488 |
| Lithuania | M | 69.99 | 29.58 | 157.23 | 0.21 | 75.42 | -413,201 | 826,409 |
| Lithuania | F | 79.99 | 30.76 | 130.82 | 0.09 | 84.63 | -387,835 | 775,676 |
| Macedonia | M | 75.06 | 25.31 | 139.34 | 0.19 | 78.82 | -385,439 | 770,884 |
| Macedonia | F | 79.01 | 25.25 | 129.31 | 0.37 | 82.35 | -373,732 | 747,471 |
| Mexico | M | 73.46 | 38.20 | 140.93 | 0.31 | 81.23 | -377,303 | 754,612 |
| Mexico | F | 79.13 | 32.09 | 142.57 | 0.25 | 84.52 | -355,415 | 710,837 |
| Netherlands | M | 81.22 | 26.81 | 134.75 | 0.14 | 84.90 | -380,419 | 760,844 |
| Netherlands | F | 85.70 | 30.47 | 127.85 | 0.27 | 89.64 | -374,490 | 748,986 |
| New Zealand | M | 82.22 | 30.33 | 128.37 | 0.34 | 86.47 | -384,245 | 768,495 |
| New Zealand | F | 85.92 | 30.25 | 127.48 | 0.17 | 89.78 | -373,705 | 747,416 |
| Norway | M | 81.72 | 30.31 | 127.45 | 0.23 | 85.97 | -384,766 | 769,538 |
| Norway | F | 85.87 | 30.21 | 126.88 | 0.29 | 89.69 | -375,400 | 750,806 |
| Poland | M | 74.04 | 26.69 | 163.83 | 0.38 | 78.36 | -406,397 | 812,801 |
| Poland | F | 82.99 | 29.43 | 132.25 | 0.36 | 87.09 | -382,782 | 765,570 |
| Portugal | M | 79.16 | 36.24 | 117.08 | 0.44 | 84.51 | -387,210 | 774,426 |
| Portugal | F | 84.84 | 33.35 | 113.51 | 0.31 | 88.58 | -362,727 | 725,460 |
| Romania | M | 70.21 | 28.59 | 151.98 | 0.39 | 75.30 | -401,343 | 802,692 |
| Romania | F | 77.97 | 29.96 | 126.46 | 0.32 | 82.45 | -382,073 | 764,153 |
| Russia | M | 61.03 | 24.28 | 226.02 | 0.40 | 64.67 | -417,356 | 834,718 |
| Russia | F | 75.84 | 33.14 | 128.40 | 0.27 | 81.43 | -396,304 | 792,614 |
| Singapore | M | 81.39 | 25.66 | 150.06 | 0.14 | 85.00 | -389,054 | 778,113 |
| Singapore | F | 86.02 | 26.55 | 143.07 | 0.10 | 89.49 | -378,248 | 756,501 |
| Slovenia | M | 76.12 | 30.06 | 127.12 | 0.60 | 80.82 | -393,222 | 786,450 |
| Slovenia | F | 83.15 | 30.09 | 117.89 | 1.43 | 86.78 | -371,341 | 742,689 |
| Slovakia | M | 72.89 | 24.99 | 156.45 | 0.33 | 76.77 | -397,338 | 794,683 |
| Slovakia | F | 81.00 | 28.90 | 123.87 | 0.15 | 84.87 | -373,410 | 746,826 |
| Sweden | M | 82.27 | 29.58 | 127.88 | 0.21 | 86.32 | -382,260 | 764,526 |
| Sweden | F | 86.05 | 29.51 | 128.18 | 0.35 | 89.77 | -375,396 | 750,798 |
| Switzerland | M | 82.76 | 30.22 | 128.79 | 0.28 | 86.95 | -382,362 | 764,730 |
| Switzerland | F | 87.18 | 30.63 | 124.78 | 0.37 | 90.87 | -367,018 | 734,042 |
| UK | M | 80.30 | 28.92 | 135.47 | 0.21 | 84.58 | -389,866 | 779,738 |
| UK | F | 84.36 | 28.79 | 133.65 | 0.14 | 88.24 | -379,629 | 759,263 |
| USA-Blacks | M | 73.39 | 29.56 | 160.82 | 0.22 | 78.62 | -411,730 | 823,465 |
| USA-Blacks | F | 80.33 | 30.50 | 151.27 | 0.26 | 85.32 | -401,532 | 803,069 |
| USA-Hispanics | M | 81.66 | 33.59 | 136.45 | 0.18 | 87.06 | -400,153 | 800,313 |
| USA-Hispanics | F | 86.22 | 29.83 | 140.36 | 0.10 | 90.39 | -384,532 | 769,071 |
| USA-Whites | M | 79.33 | 32.82 | 132.22 | 0.19 | 84.61 | -398,526 | 797,058 |
| USA-Whites | F | 83.77 | 30.22 | 135.11 | 0.15 | 88.08 | -386,454 | 772,914 |
